# SaxoForN – Transregionales allgemeinmedizinisches Forschungspraxennetz Dresden und Frankfurt am Main: Konzept einer innovativen Forschungspraxeninfrastruktur

**DOI:** 10.1007/s00103-023-03722-3

**Published:** 2023-06-13

**Authors:** Karola Mergenthal, Corina Güthlin, Astrid A. Klein, Jennifer Engler, Meike Gerber, Jenny Petermann, Steve Piller, Antje Bergmann, Ferdinand M. Gerlach, Karen Voigt

**Affiliations:** 1 Institut für Allgemeinmedizin, Goethe-Universität Frankfurt am Main, Theodor-Stern-Kai 7, 60590 Frankfurt am Main, Deutschland; 2 Medizinische Fakultät Carl Gustav Carus, Bereich Allgemeinmedizin, Technische Universität Dresden, Dresden, Deutschland; 3 Institut für Geschichte der Medizin, Technische Universität Dresden, Dresden, Deutschland

**Keywords:** Ambulante Versorgung, Primärversorgung, Klinische Studien, Versorgungsforschung, Qualifikation, Outpatient care, Primary care, Clinical trials, Health services research, Qualification

## Abstract

Etablierte allgemeinmedizinische Forschungspraxennetze (FPN) erleichtern die Durchführung von klinischen Studien sowie Studien der Versorgungsforschung im hausärztlichen Setting. Seit Februar 2020 fördert das Bundesministerium für Bildung und Forschung (BMBF) deutschlandweit den Aufbau von 6 allgemeinmedizinischen FPN und einer Koordinierungsstelle, um eine nachhaltige ambulante Forschungsinfrastruktur aufzubauen und so die Quantität und Qualität der Forschung in der Primärversorgung zu erhöhen.

Im vorliegenden Artikel wird das Konzept des „Allgemeinmedizinischen Forschungspraxennetzes Dresden/Frankfurt am Main – SaxoForN“ vorgestellt und dessen innovative Funktionsweise und Strukturen erläutert. Es handelt sich um einen transregionalen Verbund der beiden regionalen FPN mit den Namen „SaxoN“ (Dresden/Sachsen) und „ForN“ (Frankfurt am Main/Hessen), die sowohl transregional als auch lokal Forschungsprojekte umsetzen. Dafür wurden gemeinsame Standards konsentiert und harmonisierte Strukturen, z. B. zur Dateninfrastruktur, Qualifizierung, Partizipation sowie Akkreditierung, entwickelt und an beiden Standorten implementiert.

Ein kritischer Erfolgsfaktor wird sein, ob und inwiefern die Standards und Strukturen sowie die Ressourcenplanung nachhaltig gestaltet werden können, um langfristig qualitativ gute Forschung über die FPN zu ermöglichen. Dazu gehören die Akquise und langfristige Bindung der Praxen an das FPN, die Qualifizierung der Forschungspraxenteams sowie eine möglichst weitgehende Standardisierung der Prozesse, regelmäßige Dokumentation von Basisdaten und Versorgungsdaten aus den Praxen.

## Abstract (en)

Establishing primary care research networks (PCRNs) makes it easier to conduct clinical trials and health services research in a general-practice setting. Since February 2020, the German Federal Ministry of Education and Research (BMBF) has sponsored the development of six PCRNs and a coordination unit throughout Germany, with the aim of setting up a sustainable outpatient research infrastructure to raise the quantity and quality of primary care.

The present article describes the design of a PCRN in Dresden and Frankfurt am Main – SaxoForN – and explains its structure and how it operates. The network is a transregional alliance between the two regional PCRNs named “SaxoN” (Dresden/Saxony) and “ForN” (Frankfurt am Main/Hesse), both of which run transregional and local research projects. For this purpose, joint standards and harmonized structures, for example with respect to the data infrastructure, qualifications, participation, and accreditation, were agreed upon and implemented at both sites.

A critical success factor will be whether and to what extent the standards and structures, as well as resource planning, can be designed sustainably enough to permit the PCRNs to carry out high-quality research over the long term. To achieve this, the PCRNs will have to attract new practices and build up lasting relationships with them, qualify the research practices in order to standardize processes as far as possible, and regularly document their basic information and healthcare data.

## Einleitung

Um eine sichere und effektive Gesundheitsversorgung zu gewährleisten, sind eine starke ambulante haus- bzw. primärärztliche Grundversorgung der Bevölkerung sowie eine allgemeinmedizinische Forschung in diesem Setting unabdingbar [1, 2]. Diese Forschung ist dabei kein Selbstzweck, sondern unterstützt die bedarfsgerechte und effektive Versorgung sowie die Patientensicherheit. Klinische Forschung findet bislang meist in (Universitäts‑)Kliniken statt, der überwiegende Teil der Patient*innen wird jedoch ambulant und meist im primärärztlichen Setting behandelt [3, 4]. Infolgedessen waren und sind in klinischen Studien vorwiegend Betroffene mit einzelnen Krankheitsbildern, kaum Neben- oder Begleitdiagnosen und meist jüngeren Alters (< 65 Jahre) eingeschlossen. Ältere Menschen, Personen mit leichteren oder mit Mehrfacherkrankungen und solche, die sich nicht an die empfohlene Therapie halten, standen bislang selten im Mittelpunkt der Aufmerksamkeit [5]. Diese Gruppe macht jedoch den Großteil der Patient*innen im Versorgungssystem aus und fast alle gehen regelmäßig bzw. mehrfach im Jahr zu ihrer Hausärztin bzw. ihrem Hausarzt [6, 7].

Ergebnisse aus Forschungsprojekten, die an einer hochselektierten Patientengruppe durchgeführt werden und meist nur eine (schwerwiegende) Erkrankung in den Fokus nehmen, sind nur begrenzt übertragbar auf eine ältere, multimorbide, chronisch erkrankte Population. Da die Allgemeinmedizin die Disziplin ist, in der diese Patientengruppen am häufigsten gesehen werden [7], kommt ihr eine zentrale Rolle zu. Um den Herausforderungen einer alternden Gesellschaft und dem ambulanten Versorgungsbedarf von zunehmend mehr Patient*innen mit mindestens einer chronischen Erkrankung begegnen zu können, werden wissenschaftlich hochwertige klinische und versorgungsepidemiologische Studien zu allgemeinmedizinischen Fragestellungen benötigt.

Im Vergleich zu anderen Ländern fehlen aber in Deutschland regionale und überregionale Studien zu den Themen der Allgemeinmedizin. Zurückzuführen ist dies vor allem darauf, dass die Allgemeinmedizin bei der Durchführung der Studien auf das ambulante Setting angewiesen ist, in dem jedoch die bestehenden Strukturen für die Durchführung klinischer Forschung und Versorgungsforschung noch unzureichend ausgebaut sind. Hier setzt ein allgemeinmedizinisches Forschungspraxennetz (FPN) an, das durch die Etablierung geeigneter Forschungsstrukturen die Realisierung qualitativ hochwertiger Forschungsprojekte ermöglicht.

Primary Care Research Networks (PCRN) wurden in den USA [8] schon vor über 40 Jahren eingerichtet mit dem Ziel, die Rekrutierung und Teilnahme an Primärversorgungsstudien zu erhöhen und die Zusammenarbeit zwischen den ambulanten Versorger*innen und den akademisch Forschenden zu stärken [5, 9, 10]. Hierzulande wurde die akademische Basis der Allgemeinmedizin in den letzten Jahren durch die Einrichtung von selbstständigen Professuren an vielen medizinführenden Universitäten verbreitert [11], zunächst um allgemeinmedizinische Lehre an den Fakultäten sicherzustellen. Nun müssen dieser Akademisierung Strukturen folgen, die sowohl die Koordination, Organisation und Durchführung als auch die wissenschaftliche Aufarbeitung der Studien gewährleisten und den Praxen die Möglichkeit geben, versorgungsrelevante Forschungsfragen „aus der Praxis – für die Praxis“ zu generieren.

Um eine entsprechende Forschungspraxeninfrastruktur in Deutschland systematisch zu unterstützen, fördert das Bundesministerium für Bildung und Forschung (BMBF) seit 2020 den Aufbau von 6 regionalen bzw. transregionalen allgemeinmedizinischen FPN und einer Koordinierungsstelle in Berlin^1^. Zusammen bilden sie die Initiative Deutscher Forschungspraxennetze (DESAM-ForNet^2^).

Eines der geförderten FPN ist das „Forschungspraxennetz Allgemeinmedizin Dresden/Frankfurt am Main – SaxoForN“^3^, welches aus 2 lokalen FPN in Dresden/Sachsen (namens „SaxoN“) und Frankfurt am Main (namens „ForN“) besteht. In der Region Dresden (TU Dresden, Bereich Allgemeinmedizin) wird ein neues FPN aufgebaut (Ziel: 50 Praxen) und in der Region Frankfurt/Rhein-Main-Gebiet/Hessen (Goethe-Universität Frankfurt am Main, Institut für Allgemeinmedizin) ein bereits existierendes FPN ausgebaut (Ziel: 200 Praxen). SaxoForN deckt verschiedene Regionen (eine Metropolregion, städtische, ländliche, unterversorgte Regionen) in Deutschland ab und ermöglicht die Durchführung von klinischen Studien und Studien der Versorgungsforschung, die die besonderen Bedürfnisse von hausärztlichen Teams sowie Patient*innen widerspiegeln.

Im vorliegenden Artikel wird das Konzept des allgemeinmedizinischen FPN Dresden/Frankfurt am Main – SaxoForN – vorgestellt. Dessen innovative Funktionsweise wird aufgezeigt und es werden einige zentrale Strukturen und deren Bedeutung erläutert.

## Funktionsweise und Strukturen des Forschungspraxennetzes SaxoForN

**Figure 1:**
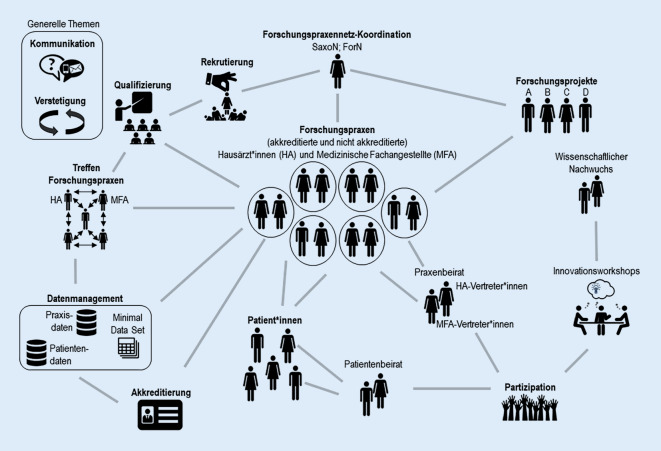

Das FPN ist so angelegt, dass die Forschungspraxen (FP) die Kernelemente bilden (Abb. 1). Für die Koordination der Vernetzungsprozesse und als Ansprechpartner*in für die lokalen FP ist in jedem FPN eine regional angesiedelte Forschungspraxennetz-Koordination etabliert. Die Rekrutierung, Qualifizierung und Akkreditierung der FP finden unabhängig von spezifischen Forschungsprojekten statt, so dass ein Pool an qualifizierten und an Forschung interessierten Praxisteams vorhanden ist, auf den unterschiedliche Forschungsprojekte dann bei Bedarf zurückgreifen können. Das „Wir-Gefühl“ im FPN wird gestärkt durch gemeinsame Veranstaltungen und Möglichkeiten zur aktiven Beteiligung an Forschung durch ein großes Angebot an partizipativen Formaten (wie Innovationsworkshops, Praxenbeirat, Patientenbeirat; siehe Abschnitt Partizipation). Durch die Kombination der Teilnahme an Forschungsprojekten und ggf. einer Akkreditierung als „Akademische Forschungspraxis“ wird die Durchführung von qualitativ hochwertigen, nachhaltigen allgemeinmedizinischen Studien sichergestellt.

### Forschungspraxen in SaxoForN – Rekrutierung, Qualifizierung und Akkreditierung

#### Rekrutierung von Forschungspraxen für das Forschungspraxennetz SaxoForN.

Die Rekrutierung von (neuen) Hausarztpraxen für das FPN erfolgt über einen multimodalen Ansatz: Durch Anschreiben z. B. über Adressdatenbanken der Kassenärztlichen Vereinigung, über frühere Projektteilnahmen und direkte Ansprache interessierter Praxen – z. B. auf Veranstaltungen – werden kontinuierlich neue FP für eine mögliche Teilnahme sensibilisiert. Die Bemühungen zielen auf Hausarztpraxen, die aus einem oder mehreren forschungsinteressierten Ärzt*innen für Allgemeinmedizin oder hausärztlich tätigen Internist*innen und 1–2 Medizinischen Fachangestellten (MFA; oder entsprechend qualifiziertem Praxispersonal) bestehen.

#### Teilnahme von Forschungspraxen in SaxoForN.

**Figure 2:**
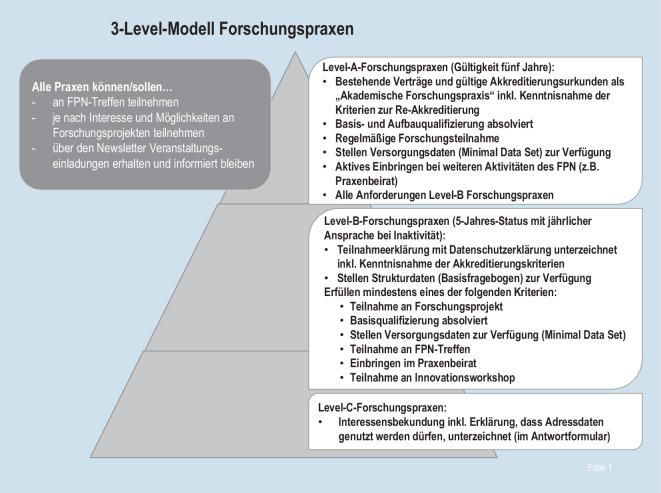

Teilnehmende FP werden in einem „3-Level-Modell“ unterschieden (Abb. 2). Um forschungsinteressierten allgemeinmedizinischen Praxen einen niedrigschwelligen Zugang zum FPN zu ermöglichen, reichen für die Aufnahme ins FPN eine Interessenbekundung und die Zustimmung zur Datenhaltung zwecks Kontaktaufnahme aus (Level-C-FP). Somit erhalten die Praxen wichtige Informationen aus dem FPN und haben die Möglichkeit, an den Veranstaltungen teilzunehmen. Sind die Praxen allerdings zu einer aktiven Teilnahme an Forschungsprojekten bereit, folgt die Übermittlung einer Teilnahmeerklärung am FPN und die Bitte um Bereitstellung von Strukturdaten zur Beschreibung der Praxis. Diese Praxen können an Basis- und Aufbauqualifizierungen, an partizipativen Veranstaltungsformaten wie Praxenbeirat oder Innovationsworkshops teilnehmen und werden um die Bereitstellung von aggregierten Versorgungsdaten gebeten (Level-B-FP). Nimmt eine FP regelmäßig an den genannten Aktivitäten des FPN teil und erfüllt alle Akkreditierungskriterien (siehe Abschnitt zur Akkreditierung der FP unten), erhält sie auf vertraglicher Basis die Möglichkeit, den Titel „Akademische Forschungspraxis“ zu führen (Level-A-FP).

Die Ansprache der Praxen (z. B. in Bezug auf neue Forschungsprojekte, Veranstaltungen) erfolgt immer über die FPN-Koordination (Abb. 1). Damit soll sichergestellt werden, dass die Praxen a) entsprechend ihrer Interessen (die sie mit dem Ausfüllen der Teilnahmeerklärung hinterlegt haben) und b) nur für allgemeinmedizinisch relevante Forschungsprojekte angefragt werden.

#### Qualifizierung der Forschungspraxen für die Teilnahme an Forschungsprojekten.

**Figure 3:**
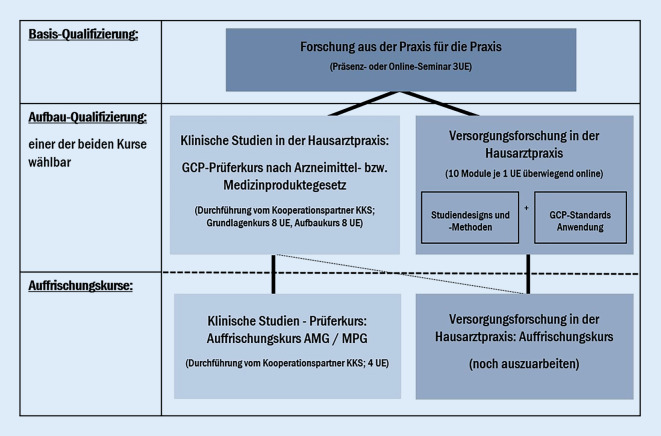

Alle FP sollen zunächst einen guten Überblick über die Qualifizierungsangebote im FPN erhalten und wahrnehmen, dass Forschung im hausärztlichen Setting sehr wichtig und interessant ist. Dafür besteht seitens des FPN das Angebot, dass alle Ärzt*innen und je Praxis bis zu 2 MFA eine sogenannte Basisschulung in Anspruch nehmen können (Abb. 3). Hierbei handelt es sich um eine 2,5-stündige Einführung in medizinische Forschung allgemein und ihre Spezifika im Bereich der Allgemeinmedizin. Es werden folgende Themen bearbeitet:

- Bedeutung und Ablauf (medizinischer) Forschung,
- Nutzen von Forschung für die einzelne Hausarztpraxis,
- Forschung im allgemeinmedizinischen FPN für die Weiterentwicklung der Patientenversorgung,
- Studientypen und -arten in der medizinischen Forschung,
- Überblick über das FPN ForN bzw. SaxoN,
- Überblick über die aktuellen Forschungsprojekte.

Auch mit dem Regelwerk zur guten klinischen Praxis (GCP-Guideline [12]) werden die Teilnehmenden vertraut gemacht.

Nach erfolgter Basisschulung können die FP wählen, ob sie den Prüferkurs nach dem Arzneimittel- bzw. Medizinproduktegesetz absolvieren möchten, den SaxoForN in Kooperation mit dem Koordinierungszentrum klinischer Studien (KKS) in Dresden^4^ anbietet, oder die Aufbauqualifizierung „Versorgungsforschung in der Hausarztpraxis“ (Abb. 3). Hierbei handelt es sich um verschiedene Module, die in einer gemeinsamen Arbeitsgruppe aller 6 geförderten FPN in DESAM-ForNet erarbeitet wurden. Themen sind u. a. Rollen und Verantwortlichkeiten, Qualitätssicherung und Qualitätskriterien, Datenschutz und Ethik, Studientypen, Entwicklung eines Fragebogens, evidenzbasierte Medizin, Lesen wissenschaftlicher Texte und Literaturrecherche. Für die teilweise unter hoher Belastung stehenden FP wurde diese Aufbauqualifizierung in 10 Module gesplittet. Sie werden teilweise in Präsenz, meist jedoch virtuell als eLearning Module durchgeführt (jeweils 1–2 Unterrichtseinheiten (UE)), so dass sie individuell bearbeitet werden können.

Während der Phase des Aufbaus von SaxoForN, d. h. während der Förderdauer bis Januar 2025, können die Praxen alle Qualifizierungsangebote kostenfrei in Anspruch nehmen.

#### Akkreditierung der Forschungspraxen.

**Figure 4:**
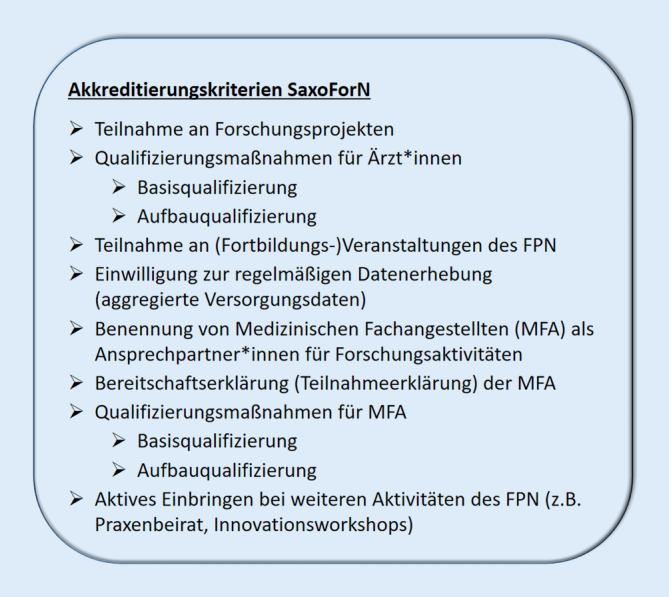

Sobald eine Praxis alle Akkreditierungskriterien (Abb. 4) erfüllt hat, wird ihr die Akkreditierung am jeweiligen Universitätsstandort angeboten. Dafür wird eine vertragliche Vereinbarung zwischen der Fakultät der jeweiligen Universität und den betreffenden Forschungsärzt*innen geschlossen. Im Vertrag wird u. a. vereinbart, dass die FP sich regelmäßig an Aktivitäten des FPN beteiligen und Ansprechpartner*innen aus den Reihen der MFA für Forschungsangelegenheiten benennen. Im Gegenzug wird den FP zugesichert, dass das FPN entsprechende Angebote zur Qualifizierung und Teilnahme an Forschungsprojekten bereitstellt. Zudem wird den FP durch den Vertrag und die Akkreditierungsurkunde bestätigt, dass sie sich als forschungsqualifiziert erwiesen haben und sie den Titel „Akademische Forschungspraxis“ tragen dürfen.

### Partizipation im Forschungspraxennetz und in Forschungsprojekten

Im FPN SaxoForN ist die Beteiligung aller Akteur*innen sowohl bei Auf- und Ausbau des FPN als auch bei der Planung und Durchführung von Forschungsprojekten umgesetzt. Hierfür wurde ein für SaxoForN passendes Partizipationskonzept ausgearbeitet [13] und im Rahmen einer Arbeitsgruppe in DESAM-ForNet weiterentwickelt [14].

Ärzt*innen und MFA, die daran interessiert sind, selbst Forschungsideen einzubringen bzw. die Ausgestaltung von Forschungsprojekten mitzugestalten, erhalten dazu die Möglichkeit im Rahmen a) des Praxenbeirates [13] oder bei b) Innovationsworkshops ([13]; Abb. 1).

Beim *Praxenbeirat* handelt es sich um eine Gruppe von interessierten Ärzt*innen und MFA. Sie werden 2‑mal pro Jahr zu Treffen eingeladen. Es finden Diskussionen statt, z. B. zu Bedarfen, Umsetzbarkeit und Machbarkeit von Forschungsprojekten und zum Aufbau des FPN. Zudem finden bedarfsorientiert Anfragen an Mitglieder dieser Gruppe statt, z. B. zur Pilotierung von Leitfäden oder von Studieninformationen, Einwilligungserklärungen oder Ergebnisbooklets. Die Teilnahme am Praxenbeirat ist für die Akkreditierung als FP anrechenbar.

Bei den *Innovationsworkshops* handelt es sich um ein ähnliches Format. Allerdings werden hier vor allem (neue) Forschungsideen diskutiert und neben Ärzt*innen und MFA auch Patientenvertreter*innen und interessierte Nachwuchswissenschaftler*innen wie Doktorand*innen oder an Forschung interessierte Ärzt*innen in Weiterbildung einbezogen [13].

Beim *Patientenbeirat* handelt es sich um eine feste Gruppe. Um keine Personen auszuschließen, wurden in die Gruppe neben hausärztlichen Patient*innen auch Bürger*innen mit und ohne Vorerkrankungen aufgenommen. An jedem Standort wurde ein Patientenbeirat etabliert. Über den Patientenbeirat wird die Sichtweise von hausärztlichen Patient*innen eingebracht, z. B. zu patientenrelevanten Fragestellungen oder der Relevanz von Forschungsergebnissen [13].

### Gemeinsam forschen im Forschungspraxennetz SaxoForN

Im FPN finden regelmäßig Treffen des gesamten FP-Teams mit allgemeinmedizinischen Forschenden statt. In diesen Treffen werden ebenfalls neue Projektideen der allgemeinmedizinischen Institute oder externen Forschungspartner*innen oder auch Ergebnisse vorgestellt und diskutiert, z. B. unter dem Aspekt der Relevanz für hausärztliche Patientengruppen oder der Machbarkeit im Praxisalltag. Diese Treffen dienen dem Austausch, der Vernetzung und fördern das „Wir-Gefühl“, d. h. die Identitätsentwicklung als Mitglied des FPN.

#### Forschungsprojekte planen und durchführen.

Alle Forschergruppen, die an den jeweiligen Institutionen unter Einbezug von Hausarztpraxen oder Patient*innen Projekte durchführen wollen, können und sollen auf das FPN zurückgreifen. Das FPN unterstützt die Rekrutierung, denn das FPN stellt einen Pool an qualifizierten FP dar, die sich explizit für allgemeinmedizinische Forschung interessieren und die sich zum Teil im Rahmen von partizipativen Methoden auch an der Ausgestaltung von Themen und Projekten beteiligt haben, d. h. einschlägige Erfahrung mitbringen. Diese Koordination und das Aufrechterhalten dieser Strukturen und Funktionen bedeuten Aufwand, der in den Projekten budgetiert werden muss.

#### Abfragen hausärztlicher Kennzahlen.

Die FP im FPN übersenden einige Praxisstrukturdaten sowie quartalsweise aggregierte Versorgungsdaten aus ihren Praxisverwaltungssystemen (PVS) an das FPN. Da aufgrund der Heterogenität der Systeme keine direkte Datenabfrage erfolgen kann, müssen diese Daten zunächst von der Praxis nachgesehen (z. B. Abrechnungsziffern) und dann händisch in ein elektronisches Erfassungssystem übertragen werden. Mit Hilfe dieser Versorgungsdaten kann die hausärztliche Tätigkeit abgebildet werden (z. B. wird nach der Anzahl der Impfungen oder bestimmter Früherkennungsleistungen gefragt). Es kann mit Hilfe dieser Daten aber auch eine Verteilung von bestimmten Diagnosen oder Interventionen analysiert werden, was Rückschlüsse auf ein mögliches Rekrutierungspotenzial für geplante Forschungsprojekte ermöglicht.

#### Kommunikation von Forschungsergebnissen.

Für die Öffentlichkeit stehen Informationen über die Website www.saxoforn.net sowie über regelmäßige Newsletter, Pressemitteilungen und Flyer bereit. Aus Forschungsprojekten entstehen neben Publikationen auch Ergebnisbroschüren in allgemeinverständlicher Sprache, die den Teilnehmenden nach Studienende zur Verfügung gestellt werden.

## Was muss für die langfristige Verstetigung des FPN beachtet werden?

Das hier beschriebene FPN SaxoForN geht auf eine Förderung des BMBF zurück, ohne die der quantitative und qualitative Auf- und Ausbau hausärztlicher FPN-Strukturen nicht oder nur sehr beschränkt möglich ist. Neben der reinen Rekrutierung von möglichen FP sind in SaxoForN flankierende Konzepte beschrieben, die 1) Praxisteams befähigen sollen, neben ihrem Arbeitsalltag qualitativ hochwertige Forschung durchzuführen, die 2) es hausärztlichen Teams und ihren Patient*innen ermöglichen, sich direkt in Forschungsprojekte einzubringen, und die 3) für eine gute Kommunikation von Forschungsergebnissen aus der hausärztlichen Praxis sorgen können.

### Ambulante Forschung auf hohem Niveau.

Mit der Etablierung dieser für Deutschland innovativen Strukturen und Prozesse kann explizit dazu beitragen werden, dass für die Allgemeinmedizin relevante Forschung in alltagsnahen Settings auf hohem wissenschaftlichen Niveau durchgeführt werden kann. Die gezielte und nachhaltige Qualifizierung der Praxisteams ist dafür unabdingbar. Mit der Basis- und Aufbauqualifizierung werden den beteiligten Praxisteams Forschungsstandards vermittelt, die eine hohe Forschungsqualität sicherstellen. Mit der Akkreditierung wird den FP die Kompetenz bescheinigt, dass sie sich als forschungsqualifiziert erwiesen haben. Die Akkreditierung ist somit ein Qualitätsmerkmal, welches die Praxen als sogenannte akademische Forschungspraxis nach außen darstellen können. Diese mögliche Außendarstellung kann eine Motivation darstellen, sich dem FPN anzuschließen [15, 16].

### Einbindung der Perspektive der Beteiligten.

Durch partizipative Formate wie den Praxenbeirat und den Innovationsworkshop wird die Expertise aller in einer FP beschäftigten und an der Versorgung beteiligten Berufsgruppen aktiv einbezogen. Der Einbezug aller Stakeholder soll sowohl die Qualität und externe Validität als auch die Legitimation von Forschung unterstützen [17]. Es ist wichtig, (immer wieder) zu vermitteln, dass Forschung und darauffolgend die Patientenversorgung von der Einbindung der Perspektive der Forschungsärzt*innen, der MFA und der Patient*innen profitiert [13]. Die Beteiligung der Zivilbevölkerung durch partizipative Formate wird von Projektförderern wie dem BMBF zunehmend forciert [17].

### Versorgungsdaten für Forschung zugänglich machen.

Während in internationalen FPN große individuelle Datensätze bzw. Registerdaten genutzt werden, um alltagsrelevante Fragestellungen in rasch zur Verfügung stehenden Abfragen zu beantworten [18, 19], existieren in Deutschland keine vergleichbaren Register. Zudem existieren hierzulande über 180 verschiedene Praxisverwaltungssysteme (PVS) im ambulanten Bereich [20], welche bislang keinen standardisierten Datenaustausch ermöglichen, so dass Versorgungsdaten aus den PVS (noch) nicht für die Forschung nutzbar gemacht werden können. So lange in Deutschland noch nicht regelhaft elektronische Patientenakten (ePA) genutzt werden, findet im SaxoForN-Projekt zunächst die Analyse von aggregierten (zusammengefassten) Versorgungsdaten statt, welche die Praxen regelmäßig ermitteln. Der Vorteil aggregierter Daten liegt u. a. darin, dass keine besonders schützenswerten gesundheitlichen Daten einzelner Personen übermittelt werden. Die Daten werden lediglich unter einer pseudonymisierten Praxiskennung in ein elektronisches Erfassungssystem eingetragen.

Gerade durch die rasche Erfassung und Bereitstellung von Daten (bspw. in Ausnahmesituationen wie der COVID-19-Pandemie) können schnell Rückschlüsse auf die Versorgungsrealität, ggf. auf Engpässe in der Versorgung, Ressourcenmängel oder -fehlallokationen oder regionale Unterschiede gezogen werden. Diese Ergebnisse können gesundheitspolitischen Entscheidungsträgern schnell gespiegelt werden.

Innerhalb der Initiative Deutscher Forschungspraxennetze (DESAM-ForNet) wird aktuell der Aufbau einer Datenbank mit den jeweiligen regionalen Netzen abgestimmt. Ziel ist der Aufbau einer transregionalen Metadatenbank, welche einerseits mittels dieser digitalen Unterstützung die bundesweiten Kooperationen bei Forschungsprojekten vereinfachen und beschleunigen soll, zum anderen die Interoperabilität mit bestehenden Festlegungen der Medizininformatik-Initiative eröffnen kann.

### Austausch auf Augenhöhe.

Eine wichtige Struktur bildet der regelmäßige Austausch der FP mit den allgemeinmedizinischen Forscher*innen. Aufgrund vorangegangener Erfahrungen mit externen Forschungsgruppen, die hausärztliche Praxen lediglich als „Dienstleister“ oder „Datenlieferanten“ in Forschungsprojekte integrieren wollten, hat sich diese Form der regelhaften Diskussion aller geplanten Projekte mit den hausärztlichen Forschungspraxen etabliert. Externe Forschungsgruppen erhalten so sehr direkt eine Rückmeldung zur Relevanz für die Allgemeinmedizin und die Möglichkeiten der Umsetzung im Praxisalltag. FP profitieren, da sie sich bei relevanten Themen einbringen können und früh neue Diagnose- oder Therapieansätze kennenlernen.

### Etablierung langfristiger Forschungsstrukturen.

Der Zeitplan für den Aufbau der FPN-Infrastruktur ist eng und durch die zusätzliche hohe Belastung der Hausarztpraxen durch die COVID-19-Pandemie ist die Rekrutierung von neuen Praxen teilweise sehr schwierig. In den Hausarztpraxen stehen nach wie vor die Organisation und Durchführungen von Infektions- und Impfsprechstunden, die vermehrten Anfragen von (verunsicherten) Patient*innen sowie die Behandlung der Infizierten, parallel zur sonstigen hausärztlichen Routineversorgung von Personen mit chronischen Erkrankungen, im Vordergrund. Zudem ist die Teilnahme der Praxen im FPN mit keinem finanziellen Ausgleich für den Aufwand verbunden. Die FP erhalten jedoch eine Aufwandsentschädigung für Forschungs- und Praxenbeiratstätigkeit.

Der Förderzeitraum umfasst die Jahre 2020 bis Januar 2025. In dieser Zeit ist vor allem der Strukturaufbau zu leisten und die Entwicklung als FPN zu gestalten. Am Ende der Strukturförderung soll dann eine große Gruppe an motivierten, qualifizierten, partizipativ eingebundenen FP-Teams existieren, die bei Bedarf ad hoc Forschungsprojekte durchführen können. Das FPN soll auch Unterstützung für Forschungsprojekte bieten, indem dafür auf eingespielte Abläufe, Standard Operating Procedures (SOPs), Templates für Projektmaterialien etc. zurückgegriffen werden kann.

Allerdings können die Strukturen des FPN SaxoForN nur dauerhaft erhalten werden, wenn sie von Anfang an nachhaltig, insbesondere ressourceneffizient, gestaltet werden. Die aufbauenden Standorte sollten die Strukturen konsequent für Nachfolgeprojekte und zukünftige Forschungsprojekte nutzen, Ressourcen für die Nutzung planen und so zum Erhalt der Kompetenzen und des Wissens für die nachhaltige allgemeinmedizinische Forschung beitragen. Dafür werden innerhalb der Laufzeit Kostenkalkulationen entwickelt, die notwendige Budgets innerhalb zu beantragender Forschungsprojekte transparent darlegen können. Bei der Antragstellung sollte demgemäß die Nutzung der FPN-Strukturen und -Funktionen (Rekrutierung, Qualifizierung, Projekt‑/Datenbankverwaltung) finanziell abgebildet werden (z. B. in Form von Personalstellenanteilen).

Weitere „kostenpflichtige“ Leistungen und Unterstützungsangebote durch die Personen der FPN-Koordination sind denkbar (z. B. Teilnahmegebühren für Qualifizierungsmodule, Schulungsangebote für Projektmitarbeitende, Methodenberatung zur Forschung in der Primärversorgung, Beratung durch die FPN-Patientenbeiräte).

Eine weitere Möglichkeit, um Ressourcen für den Erhalt des FPN sicherzustellen, stellt die Kooperation des FPN mit externen Forschungspartner*innen aus anderen Universitäten oder anderen wissenschaftlichen Disziplinen dar. Alle externen Partner*innen, öffentlich finanziert, wie die Medizininformatik-Initiative (MII), das Robert Koch-Institut (RKI) oder Stiftungen etc., werden darauf hingewiesen, dass bereits im Antragsverfahren eine Kooperation mit dem FPN zu suchen ist. Eine solche Kooperation garantiert die Einhaltung von Qualitätsstandards und die Sicherstellung der Machbarkeit von Studien in der hausärztlichen Praxis, da das für Forschung mit Hausarztpraxen notwendige Methodenwissen direkt in die Projektentwicklung einbezogen werden kann. Auch Projektpartner*innen in Forschungskooperationen müssen Ressourcen wie standardmäßige Incentives für Praxen und administratives Personal des FPN in Anträgen mitbedenken.

### Zusammenarbeit mit anderen FPN.

In der Initiative DESAM-ForNet findet ein regelmäßiger Austausch mit den anderen geförderten FPN statt. In den Arbeitsgruppen wird netzübergreifend an der Weiterentwicklung gemeinsamer Themen gearbeitet, wie z. B. einheitliche Praxisstruktur- und soziodemografische Datenerhebungen bei allen Forschungspraxen, Entwicklung eines gemeinsamen Lernzielkatalogs für die Qualifizierungsmaßnahmen und die Entwicklung eines Research-ready-Konzeptes.

### Zusammenarbeit mit weiteren Institutionen im Gesundheitswesen.

In SaxoForN finden erste Projekte bzw. erste Gespräche mit Partner*innen im Gesundheitswesen statt, wie mit der Medizininformatik-Initiative (MII) und den Datenintegrationszentren (DIZ), dem Robert Koch-Institut (RKI) und dem Netzwerk Universitätsmedizin (NUM). SaxoForN hat Grundsätze entwickelt, die eine Zusammenarbeit auf Augenhöhe forcieren.^5^ Dazu gehört u. a., dass Forschungsfragen die Bedarfe der hausärztlichen Patientengruppen adressieren, dass die Praxen nicht nur als Datenlieferanten betrachtet werden und dass das FPN bei der Entwicklung des Studiendesigns – unter Einhaltung partizipativer Aspekte – einbezogen wird.

### Kontinuierliche Evaluation.

Der Aus- und Aufbau der unterschiedlichen Strukturen und Prozesse des FPN wird in einem iterativen Prozess kontinuierlich evaluiert und adaptiert mit dem Ziel, die Strukturen langfristig zu implementieren. Dafür wurde z. B. nach den ersten Sitzungen der Patientenbeirat interviewt (z. B. was besonders gut gefallen oder gefehlt hat oder zu Anregungen zur Gestaltung kommender Treffen) oder nach den ersten KKS-Qualifizierungsmodulen wurde quantitativ und qualitativ u. a. die Passfähigkeit für hausärztliche Praxisteams evaluiert. So konnten die Angebote – im Sinne des Qualitätsmanagements – an die Bedarfe angepasst werden.

Für einen erfolgreichen langfristigen „Betrieb“ des FPN sind folgende Voraussetzungen notwendig:

- (akkreditierte) FP in ausreichender Quantität, Qualifizierung, Motivation und mit ausreichendem Informationsfluss,
- FPN mit Nutzungsregeln, ausreichender Finanzierung und personeller Kapazität, funktionierender IT-Infrastruktur,
- ausreichende personelle Kapazitäten zur Kontaktpflege des FPN,
- Kommunikation zwischen Forschungsprojektleiter*innen und der FPN-Koordination.

### Herausforderungen.

Aufgrund der COVID-19-Pandemie kam es zu einem deutlichen Anstieg der Arbeitsbelastung für Mitarbeiter*innen der Hausarztpraxen, was die Rekrutierung von neuen FP erschwerte. Dennoch finden sich grundsätzlich interessierte Praxen, die sich in kleineren Projekten engagieren. Nach gut der Hälfte der Förderperiode konnten erste netzübergreifende Kooperationen und Antragsverfahren angebahnt werden. Doch allein über die Einwerbung neuer Forschungsprojekte kann die oben aufgezeigte Forschungspraxeninfrastruktur nicht aufrechterhalten werden. Für eine langfristige Sicherstellung der FPN sind projektunabhängige personelle und finanzielle Ressourcen unabdingbar. Ebenfalls unabdingbar für ein Gelingen relevanter Forschung ist die Überbrückung der Sektorengrenzen zwischen ambulanter und stationärer Versorgung; eine Ausweitung der Datenaufnahme aus dem stationären Kontext um Daten aus der ambulanten Versorgung könnte z. B. durch eine stärkere Kooperation mit der Medizininformatik-Initiative oder dem Netzwerk Universitätsmedizin gelingen.

## Fazit

Insgesamt werden die Strukturen und Prozesse in SaxoForN so angelegt, dass langfristig, d. h. über die Förderdauer hinaus, hochwertige und relevante allgemeinmedizinische Forschung ermöglicht werden kann. Dafür ist es essentiell, dass bereits bei der Konzeptentwicklung die langfristige Verstetigung der FPN mitgedacht wird, um die Nachhaltigkeit der entstehenden Strukturen der FPN über die Förderphase hinaus zu gewährleisten.

## Footnotes

1 https://www.gesundheitsforschung-bmbf.de/de/aufbau-einer-netzwerkstruktur-fur-forschungspraxen-11238.php (letzter Zugriff 07.05.2023).

2 https://www.desam-fornet.de/ (letzter Zugriff 07.05.2023).

3 https://www.saxoforn.net/ (letzter Zugriff 07.05.2023).

4 https://www.kks-netzwerk.de/netzwerk/ueber-uns/mitglieder/kksdresden/ (letzter Zugriff 07.05.2023).

5 https://www.saxoforn.net/forschungskooperationen (letzter Zugriff 07.05.2023).
